# Effects of maternal immune activation in porcine transcript isoforms of neuropeptide and receptor genes

**DOI:** 10.31083/j.jin.2021.01.332

**Published:** 2021-03-30

**Authors:** Bruce R. Southey, Pan Zhang, Marissa R. Keever, Haley E. Rymut, Rodney W. Johnson, Jonathan V. Sweedler, Sandra L. Rodriguez-Zas

**Affiliations:** 1 Department of Animal Sciences, University of Illinois at Urbana-Champaign, Urbana, 61801 IL, USA; 2 Illinois Informatics Institute, University of Illinois at Urbana-Champaign, Urbana, 61801 IL, USA; 3 Neuroscience Program, University of Illinois at Urbana-Champaign, Urbana, 61801 IL, USA; 4 Department of Chemistry, University of Illinois at Urbana-Champaign, Urbana, 61801 IL, USA; 5 Department of Statistics, University of Illinois at Urbana-Champaign, Urbana, 61801 IL, USA

**Keywords:** Maternal immune activation, Neuropeptide, Autism, Alternative splicing

## Abstract

The prolonged effects of maternal immune activation in response stressors during gestation on the offspring’s molecular pathways after birth are beginning to be understood. An association between maternal immune activation and neurodevelopmental and behavior disorders such as autism and schizophrenia spectrum disorders has been detected in long-term gene dysregulation. The incidence of alternative splicing among neuropeptides and neuropeptide receptor genes, critical cell-cell signaling molecules, associated with behavior may compromise the replicability of reported maternal immune activation effects at the gene level. This study aims to advance the understanding of the effect of maternal immune activation on transcript isoforms of the neuropeptide system (including neuropeptide, receptor and connecting pathway genes) underlying behavior disorders later in life. Recognizing the wide range of bioactive peptides and functional receptors stemming from alternative splicing, we studied the effects of maternal immune activation at the transcript isoform level on the hippocampus and amygdala of three-week-old pigs exposed to maternal immune activation due to viral infection during gestation. In the hippocampus and amygdala, 29 and 9 transcript isoforms, respectively, had maternal immune activation effects (*P*-value < 0.01). We demonstrated that the study of the effect of maternal immune activation on neuropeptide systems at the isoform level is necessary to expose opposite effects among transcript isoforms from the same gene. Genes were maternal immune activation effects have also been associated with neurodevelopmental and behavior disorders. The characterization of maternal immune activation effects at the transcript isoform level advances the understanding of neurodevelopmental disorders and identifies precise therapeutic targets.

## 1. Introduction

The activation of the immune system in the gestating female triggered by infection or other stressors elicit signals that can reach the developing fetus and can cause prolonged disruption of molecular pathways in the offspring’s brain [1–4]. The relationship between maternal immune activation (MIA) and neurodevelopmental disorders, such as autism and schizophrenia spectrum disorders, and other neurodegenerative disorders such as Alzheimer’s disease, has been well documented [5–7].

Neuropeptides, hormones and neuropeptide receptors participate in biological processes underlying behavior and memory [8–12], and some neuropeptides such as insulin-like growth factor 1 (IGF1) have been associated with MIA-related disorders such as autism spectrum disorders [13, 14]. Our analysis of gene-level expression profiles in the amygdala of pigs exposed to viral infection during gestation exposed the effect of MIA on some members of the neuropeptide system, including the prohormone genes proenkephalin (PENK) and proopiomelanocortin (POMC), and receptor genes corticotropin-releasing hormone receptor 2 (CRHR2), parathyroid hormone 1 receptor (PTH1R), and vasoactive intestinal peptide receptor 2 (VIPR2) [15]. Despite the established role of neuropeptide and associated receptors on cell signaling and participation on pathways associated with social, feeding and aggression behavior, evidence gathered at the gene-level of expression has been limited. Bioactive neuropeptides result from complex transcription and translation processes [16, 17]. We hypothesize that MIA may have a varying effect on the products alternatively transcribed from neuropeptide genes. Therefore, analysis at the gene-level may hinder the exposition of MIA effects and may hinder the detection of antagonistic effect at the isoform level. Furthermore, the effect of MIA on the transcript isoform profiles may vary across brain regions involved in behaviors.

The amygdala and hippocampus brain regions are part of the limbic system and modulate behavior, emotion and memory. Amygdala and hippocampus processes have been associated with social behavior [18, 19], and disruption of the amygdala has been proposed to underlie autism spectrum disorders [20, 21]. Despite the established association between behavior disorders and MIA and between behavior disorders and neuropeptide systems, disruptions of the neuropeptide systems associated by MIA in the hippocampus and amygdala are only partially understood.

The objective of the present study is to advance the understanding of transcript isoform dysregulation in the neuropeptide system caused by MIA on two brain regions that regulate behavior. The characterization of the MIA effect at the transcript isoform level will complement reports from MIA studies of neurodevelopmental disorders at the gene level, and assist in the detection of potentially antagonistic effects of MIA on isoforms within a gene. A pig model of viral-elicited MIA was used to assess the impact of the immune response during gestation on the hippocampus and amygdala neuropeptide transcriptome. The supporting objective included the identification of MIA effects on the neuropeptide transcriptome that were brain region-dependent. Neuropeptide network reconstruction and visualization enabled us to uncover interrelationships between the neuropeptide genes impacted by MIA.

## 2. Materials and methods

### 2.1 Data used

A study of the impact of MIA on the transcript isoforms was undertaken in recognition that neuropeptides and their receptors play an essential role in various physiological functions. Yet, the activity of these molecules depends on the transcribed isoform. Transcript isoform profiles from neuropeptide, corresponding receptors and genes connected through protein-protein interactions were studied in previously unpublished hippocampus samples and complemented with published amygdala samples [15] from pigs exposed to MIA relative to controls. This porcine model is suitable for MIA due to the many similarities to human physiology and the immune system [22]. The Porcine Reproductive and Respiratory Syndrome virus used to expose pigs to MIA belongs to the *Arteriviridae* family within the *Nidovirales* order, and this order includes the *Coronaviridae* family [23]. Viruses from these families and order have high incidence, causing colds and severe respiratory tract infections in human populations. These viruses can elicit immune activation in gestating females, and can cause significant financial cost to the swine industry due to reproductive failure and respiratory disease [24].

The experimental design and animal care protocols were approved by the Institutional Animal Care and Use Committee (IACUC) at the University of Illinois and were previously described [15, 25]. Briefly, pregnant gilts were intranasally inoculated with the Porcine Reproductive and Respiratory Syndrome virus (5 mL of 1 × 10^5^ TCID_50_, strain P129-BV, Purdue University, West Lafayette, IN, USA) on day 76 of gestation to induce MIA. Control gilts were inoculated with sterile Dulbecco’s modified Eagle medium on the same day. The viral inoculation protocol elicits MIA characterized by elevated body temperature and reduced feed intake and recovery two weeks after inoculation [25]. The viral-inoculated gilts received maximum daily feeding daily, and the control gilts were fed an amount equal to that consumed by immune-challenged gilts the day before. After farrowing at approximate gestation day 113, the offspring remained with their mothers and nursed, until an equal number of female and male pigs were anesthetized and euthanized at 22 days of age as previously described [15]. The hippocampus and amygdala were recognized following anatomical markers in the pig brain atlas [26] and removed, and immediately flash frozen on dry ice.

For each brain region, RNA was isolated using EZNA isolation kit following the manufacturer’s instructions (Omega Biotek, Norcross, GA, United States). The hippocampus and amygdala RNA integrity was at least 7.5 and the RNA-Seq libraries were prepared with TruSeq Stranded mRNAseq Sample Prep kit’ (Illumina Inc, San Diego, CA). The individual pig libraries of the hippocampus (n = 48) and amygdala (n = 24) were sequenced in two lanes on a NovaSeq 6000 resulting in 150 nt long paired-end reads. Prior analysis of the amygdala expression patterns at the gene level identified differential abundance on a handful of neuropeptide and receptor genes, yet insufficient to support the enrichment of the Gene Ontology neuropeptide signaling pathways [15]. The present study broadens our previous gene-level analysis into the transcript isoform profiles in the amygdala and the hippocampus, another brain region that modulates behavior. We found that transcript isoforms provided more accurate gene networks than gene-level abundance [27].

### 2.2 Data analysis

The hippocampus and amygdala RNA-seq sequence reads were mapped to the *Sus scofa* genome v11.1 genome using Kallisto v0.43.0 with default settings [28]. Previously unpublished transcript isoform from neuropeptide and neuropeptide receptor genes from the hippocampus and amygdala were identified and analyzed. For each region, the count of each transcript was analyzed using a negative binomial generalized linear model of MIA, sex and the interaction between MIA and sex. The statistical analyses were conducted using SAS v9.4 (SAS Institute Inc., Cary, NC, United States). A preliminary analysis of the transcript isoforms corresponding to 185 neuropeptide and receptor genes demonstrated that less than 5% presented significant interaction effects, and therefore interaction effects are not reported. Of interest was the effect of MIA on the transcript isoform profiles, irrespectively of the sex; therefore, only the estimates of the effect of MIA, adjusted for sex effects, are presented.

Protein-protein and other molecular interaction databases were searched for genes known to connect neuropeptides and receptors that had transcript isoforms impacted by MIA in each brain region using the BisoGenet plugin [29] in the Cytoscape environment [30]. The visualization of the molecular relationships employed a maximum of two connections between neuropeptide-related genes based on the human gene and protein interactions identified by BisoGenet. Transcript isoforms of connecting pathway genes were analyzed for MIA effects using the same neuropeptide and receptor genes.

## 3. Results

### 3.1 Overall transcript isoform patterns in the hippocampus and amygdala

The hippocampus study yielded 207 transcript isoforms identified from 97 neuropeptide genes, 196 transcript isoforms from 87 neuropeptide receptor genes, and 211 transcript isoforms from 41 connecting pathway genes. Significant MIA effects (*P*-value < 0.01) were detected in 12, 9, and 8 neuropeptide, neuropeptide receptors, and connecting pathway gene transcript isoforms. Only IGF1 and phospholipase C gamma 1 (PLCG1) had two significant transcript isoforms in the hippocampus. Supplementary Table 1 lists the differential expression between treatments of all transcript isoforms and brain regions.

The amygdala study yielded 207 transcript isoforms identified from 97 neuropeptide genes, 196 transcript isoforms from 87 neuropeptide receptor genes, and 178 transcript isoforms from 49 connecting pathway genes. Significant MIA effect (*P*-value < 0.01) were detected in 3, 4, and 2 neuropeptides, neuropeptide receptors, and connecting pathway transcript isoforms, respectively, in the amygdala. No gene had more than one significant transcript isoform in the amygdala.

**Fig. 1.**
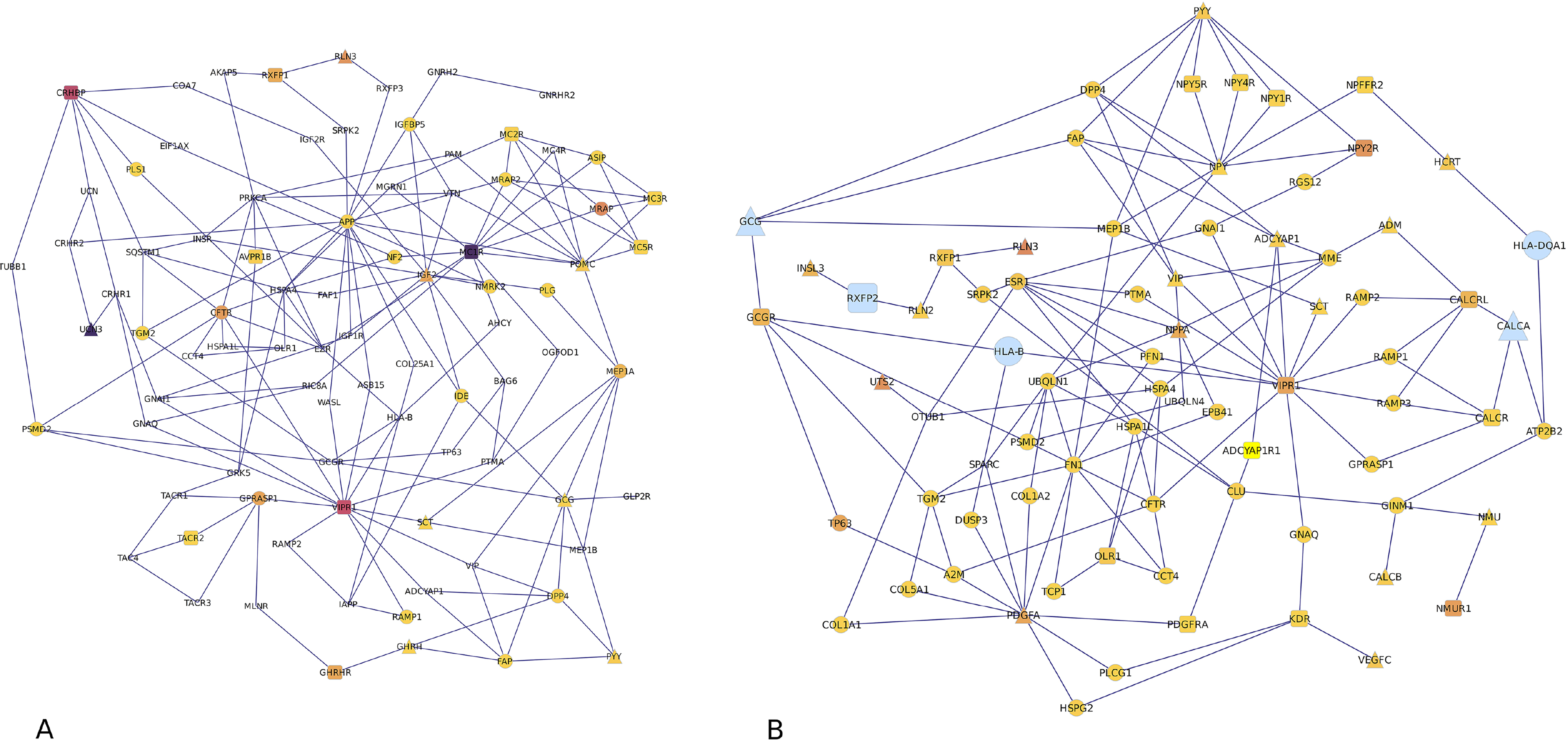
Pathway of significantly differently expressed neuropeptide (triangle), receptor (square) and connecting (circle) genes in the A) amygdala and B) hippocampus. Gene symbol size represents the transcript isoform fold change between MIA and control pigs, and gene symbols with darker colors more significantly differential expression of transcript isoform than gene symbols with lighter colors.

Table 1 summarizes the number of transcript isoforms and genes differentially expressed (*P*-value < 0.01) between MIA and control pigs within the brain region. Transcript isoforms from oxidized low-density lipoprotein receptor 1 (OLR1) and peptide YY (PYY) were significantly differentially expressed in MIA in both brain regions. Fig. 1 depicts the network of related neuropeptide, neuropeptide receptor, and connecting pathway genes in the hippocampus and amygdala, respectively.

### 3.2 Maternal immune activation effect on transcript isoform expression

The evaluation of the patterns of transcript isoform expression offered further insights into the impact of MIA. Tables 2 and 3 summarize the transcript’s hippocampus profiles isoforms over- and under-expressed in MIA relative to control pigs (*P*-value < 0.01), while Table 4 reports matching information amygdala profiles.

In the hippocampus, 19 transcript isoforms were over-expressed in MIA relative to control pigs at (Table 2), whereas 8 transcript isoforms were under-expressed in MIA relative to control pigs at *P*-value < 0.01 (Table 3). In the amygdala (Table 4), 4 transcript isoforms that were over-expressed in MIA relative to control pigs, whereas 5 transcript isoforms were under-expressed in MIA relative to control pigs at *P*-value < 0.01.

In general, differentially expressed transcript isoforms (*P*-value < 0.01) from the same gene followed the same pattern between treatment groups, and this pattern was consistent with that of other non-significant transcript isoforms from the same gene (Supplementary Table 1). Transcript isoforms that presented the same pattern in both brain regions but varying significance (*P*-value < 0.10) included calcitonin-related polypeptide 1 (CRSP1), gonadotropin-releasing hormone (type 2) receptor 2 (GNRHR2), neuropeptide Y receptor Y1 (NPY1R), OLR1, pro-melanin concentrating hormone (PMCH), PYY, solute carrier family 40 member 1 (SLC40A1), and vasoactive intestinal peptide receptor 1 (VIPR1). Transcript isoforms from arginine vasopressin receptor 1B (AVPR1B), chromogranin B (CHGB), and dipeptidyl peptidase 4 (DPP4) presented the different expression profiles across brain regions but varying significance (*P*-value < 0.10).

## 4. Discussion

The immune response of the mother to infection during gestation can impact the developing fetus and have long-lasting effects on the offspring after birth. The association between MIA and social behavior disorders such as autism spectrum disorders and schizophrenia spectrum disorders have been established in humans and biomedical models [31–33]. Pigs from gilts inoculated with the viral challenge of PRRSV during gestation exhibited lower sociability and locomotor activity compared to pigs from control gilts [25, 34–36]. The previous MIA effects are related to the susceptibility of developing brain regions that modulate behavior such as the hippocampus and amygdala. A gene expression study in the hippocampus of pig fetuses exposed to MIA detected increase the expression of three cytokines [37]. Our prior study of gene expression levels in the amygdala uncovered a few members of the neuropeptide system (POMC, VIPR2, PENK, PTH1R, CRHR2) that were affected by MIA [15]. The previous studies confirm that MIA can impact the expression levels of genes associated with behavior. However, the limited changes detected at the gene level and the multiple transcript isoforms that genes in the neuropeptide system can produce prompt us to investigate the impact of MIA at the transcript isoform level in the hippocampus and amygdala. Limitations in previous analyses have been addressed in the present study.

### 4.1 Transcript isoform patterns of maternal immune activation

The present study validated our working hypothesis that a significant mode of action of MIA is through disruption of the levels of transcript isoform expression, which in turn influences the function and activity of molecules in the neuropeptide system. Our systematic study of over 200 transcript isoforms produced by genes in the neuropeptide system enabled us to expose various MIA effects within gene and across brain regions that are subsequently exemplified. Transcript isoforms were identified in the hippocampus and hypothalamus for most neuropeptide and receptor genes known in mammals [16, 17, 38, 39]. Transcript isoforms from neuropeptide Y receptor Y6 (NPY6R), insulin (INS) and urocortin 2 (UCN2) were identified in fewer than 5% of the pigs in both regions. Transcript isoforms from neuropeptides glucagon (GCG), urocortin 3 (UCN3), and urotensin 2B (UTS2B) were identified in the hippocampus of fewer than 5% of the pigs but were identified in a higher percentage of the amygdala samples. Similarly, transcript isoform from insulin-like 3 (INSL3), secretin receptor (SCTR) and urotensin 2 (UTS2) were identified in the amygdala of fewer than 5% of the pigs. Still, it was identified in a higher percentage of the hippocampus samples. These genes with low transcript isoform numbers also have very low or no expression in the human brain [40].

#### 4.1.1 Inconsistent MIA effects on isoforms from the same gene

Our study at the transcript isoform level enabled the detection of strong MIA effects that could be obscured in analyses at the gene level when the transcript isoforms from the same gene are differentially affected by MIA. This is the case with two transcript isoforms of PYY that were differentially expressed in the same direction in both brain regions but with different significance levels. Neuropeptide PYY has been associated with feeding patterns, and the comorbidity of autism spectrum disorders is alterations in feeding behavior [41]. The detection of significant MIA effects on the patterns of isoforms from neuropeptide gene PYY offers fresh insights into these molecules’ role in MIA-associated disorders not previously established.

The original MIA-isoform associations detected in the present study can be corroborated by reports of gene associations with MIA comorbidities. PYY influences appetite, and the PYY transcript isoforms detected in the present study contain the expected active PYY peptide sequence that is conserved across vertebrates [42]. The predicted PYY transcript isoform sequence (XM_021066091.1) has a more extended reading frame than the curated sequence (NM_001256528.1), explaining the different magnitude of the MIA effect on the two transcript isoforms.

Another example of the innovative insights into the impact of MIA gained by the study of transcript isoform patterns is the receptor VIPR1 that had one transcript isoform over-expressed and another transcript isoform under-expressed in the amygdala of MIA relative to control pigs. The distinct impact of MIA across transcript isoforms may have hindered the ability of studies to detect the role of VIPR1 in MIA; however, our finding is endorsed by reports of significant differential expression of VIPR1 in the amygdala of rat lines selected for high or low ethanol consumption. This condition can disrupt inflammatory signals [43]. Similarly, transcript isoforms that presented opposite MIA profiles in the same region included GNRHR2 and ADCYAP receptor type I (ADCYAP1R1), relaxin/insulin-like family peptide receptor 1 (RXFP1) in the hippocampus and IGF1 in the amygdala.

Among the genes, including transcript isoforms, presented opposite MIA effects across brain regions G protein-coupled receptor 182 (GPR182) and connecting gene MHC class I antigen 2 (SLA-2). Although limited information is available on the role of the previous genes in MIA, our findings are corroborated by reports that GPR182 regulates the leukotriene biosynthesis pathway [44], and leukotriene are lipid mediators involved in immune response [45]. SLA-2 also participates in the immune response, and associations with MIA-associated autism have been published [46, 47].

Our investigation of transcript isoforms from immune-related genes uncovered the over-expression of SLA-2 and under-expression of GPR182 in the amygdala of MIA relative to control pigs. This finding suggests that immune activation during development can have a prolonged effect on the modulation of immune responses to stressors later in life. This result is supported by MIA pigs’ differential behavior relative to controls when injected with the immunostimulant Poly (I: C) at two months of age [25]. The opposite patterns of transcript isoforms in the previously reviewed genes reinforce the need to study the effect of inflammation signals during gestation on the offspring transcriptome later in life. The study at the gene level would cancel the distinct profiles and result in an apparent null effect of MIA on this receptor.

#### 4.1.2 Consistent MIA effects on isoform from the same genes within the brain region

Transcript isoforms of several neuropeptides, neuropeptide receptors, and connecting pathway genes in the neuropeptide system were differentially expressed (*P*-value < 0.01) in either both or one brain region. In some cases, the same transcript isoform was differentially expressed in both regions. In contrast, different transcript isoforms or isoforms from homolog genes were differentially expressed in both regions. Notable findings are the over-expression in MIA relative to control pigs for the transcript isoforms of CRSP1 and SLC40A1 in the hippocampus and amygdala. Whereas the fold change of CRSP1 was similar across regions, the fold change, the fold change of SLC40A1 was more pronounced in the hippocampus. The detection of the CRSP1 gene in the hippocampus is consistent with reports that the CRSP1 form is more abundant, and the calcitonin receptor-stimulating peptide 2 (CRSP2) and calcitonin receptor-stimulating peptide 3 (CRSP3) genes in the hippocampus of pigs [48].

Similarly, the differential expression of SLC40A1 is consistent with reports that MIA in rats disrupts the transport of iron through the materno-fetal interface, and the iron exporter ferroportin/SLC40A1 is essential for iron homeostasis. The resulting hypoferremia altered dopamine function in the adult offspring in rats and is associated with schizophrenia spectrum disorders [49]. These results demonstrate the added value of studying the impact of MIA in neuropeptide systems at the transcript isoform level.

#### 4.1.3 Consistent MIA effects on transcript isoform profiles across brain regions.

The effects of MIA on the transcript isoforms produced by PLCG1, IGF1 and neuropeptide Y (NPY) were similar in the hippocampus and amygdala. Two PLCG1 transcript isoforms and two IGF1 transcript isoforms were over-expressed in the hippocampus of MIA relative to control pigs. Two NPY transcript isoforms in the amygdala and one of them detected in the hippocampus were under-expressed in MIA relative to control pigs. The detected effect of MIA on PLCG1 is consistent with reports that PLCG1 participates in the interactome of schizophrenia [50]. Genetic deletions of PLCG1 in mice exhibited behavioral alterations, including hypoactivity and reduced anxiety [51]. The NPY transcript isoform profile detected in this study is also consistent with reports that the expression of NPY (a member of the neuroactive ligand-receptor interaction pathway) was altered in the dorsolateral prefrontal cortex of individuals with schizophrenia [52]. Likewise, IGF1 was identified in association with the risk to develop neuropsychiatric and neurodevelopmental disorders due to maternal immune activation secondary to influenza infection [53]. Our results are first to elucidate the pattern of transcript isoforms associated with MIA. Our results confirm the need to study neuropeptide and receptor mRNA abundance at the transcript isoform level. Studies at the gene level fail to account for variation between transcript isoforms.

### 4.2 Transcript isoform characterization of maternal immune activation effects

In addition to our transcript isoform-level discoveries, we confirmed reports of an association between numerous genes and MIA or MIA-related disorders. Many of the transcript isoforms presenting significant MIA effects are annotated to genes in Tables 2–4 that have differentially expressed genes between MIA and control pigs have been associated with neurological disorders. Some MIA associations with genes detected in the present study were also established at the DNA variant level. For example, single nucleotide polymorphism in or near ADCYAP receptor type I (ADCYAP1R1), NPS receptor (NPSR1), estrogen receptor 1 (ESR1), SRSF protein kinase 2 (SRPK2) genes have been associated with autism spectrum disorders, Alzheimer’s disease behavioral traits, anxiety, impulsivity and attention deficit-related traits [21, 54–59].

Among the MIA effects bridging the hippocampus and amygdala, our results offer original evidence that MIA can disrupt the vasoactive intestinal peptide (VIP) and pituitary adenylate cyclase-activating peptide (PACAP). Significant effects of MIA were detected on transcript isoforms of the neuropeptide receptor ADCYAP1R1 and vasoactive intestinal peptide receptor 1 (VIPR1) receptors and the adenylate cyclase-activating polypeptide 1 (ADCYAP1) gene that produces PACAP27 and PACAP38 neuropeptides. However, no differential expression was detected with the VIP ligand and VIPR2. While the ADCYAP1R1, VPAC1, and VPAC2 interact with receptor-activity-modifying proteins (RAMPs) [60, 61], none of the RAMPs were differentially expressed. The VIP/PACAP signaling pathway is important in brain development, psychiatric illness and behavior [62, 63]. Altered behavior was observed in mouse lines knockout for ADCYAP1 and VIP prohormone genes [64, 65], and abnormal social behaviors have been recorded in ADCYAP1R1 deficient mice [66, 67]. ADCYAP1R1 is a member of the circadian entrainment pathway with multiple transcript isoforms presenting different ligand binding properties and signal transduction, therefore modulating the response to a vasoactive intestinal peptide (VIP) and PACAP [68, 69]. Comparison of the pig ADCYAP1R1 protein sequences identified that the different transcripts corresponded to the third intercellular loop that generate differential responses to PACAP-27 [70]. Compared to normal zebrafish (*Danio renio*), larvae lacking the hop exon had increased anxiety-like behavior [71, 72]. ADCYAP1 is a member of the circadian entrainment pathway and considered a potential target for therapies to ameliorate drug abuse, migraine, stress-related psychopathologies and inflammatory diseases [69, 73–78]. In addition to the anti-inflammatory effect of VIPR1 [64], mutant mouse strains with inactivated VIPR1 showed a hippocampus-dependent deficit in fear conditions amygdala amygdala-dependent fear conditioning [79].

Peptidylglycine alpha-amidating monooxygenase (PAM) and ESR1 are genes connecting MIA-dysregulated neuropeptides and receptor transcript isoforms in the networks depicted in Fig. 1. PAM is responsible for the post-translational amidation of neuropeptides that can affect bioactivity, and this monooxygenase [80, 81] was over-expressed in MIA compared to control pigs in the hippocampus and amygdala. Our results are consistent with a report that PAM was over-expressed in the neocortex of 56-day old mice prenatally exposed to influenza [82]. ESR1 served as a connector between neuropeptides genes natriuretic peptide A (NPPA) and platelet-derived growth factor subunit A (PDGFA) and receptors RXFP1, VIPR1, and neuropeptide Y receptor Y2 (NPY2R) and was over-expressed in MIA relative to control pigs in the hippocampus and amygdala. Sequence variants of ESR1 have been linked to impaired social interaction [58, 83].

A review of the impact of MIA in the hippocampus indicates significant effects on the transcript isoforms from genes, including neuropeptide arginine vasopressin (AVP), calcitonins, hypocretin neuropeptide precursor (HCRT), IGF1, MAS related GPR family member X2 (MRGPRX2), PLCG1, relaxin 3 (RLN3), and SLC40A1. The detection of MIA associations with the previously enumerated transcript isoforms agrees with findings on behavior disorders, including reports that HCRT regulates sleep, feeding behavior, wakefulness, emotion, and stress response [41]. Similarly, RLN3 influences feeding behavior, stress responses, anxiety and memory [84], and AVP is a regulator of social behavior [85,86]. IGF1 has been associated with autism spectrum disorder symptoms [87–89], with IGF1-treatment proposed as an autism therapeutic intervention [88].

The over-expression of SLC40A1 in the hippocampus of MIA relative to control pigs suggests that transcript isoform dysregulation may be a neuro-inflammatory response to the MIA signaling [89]. SLC40A1 has been implicated in the incidence of autism spectrum disorders [90]. MRGPRX2 is a receptor with multiple neuropeptide ligands, including cortistatin-14, and this receptor’s role was noted in a review of molecular mechanisms of autism spectrum disorder [91].

Whereas two calcitonin genes, calcitonin related polypeptide alpha (CALCA) and calcitonin related polypeptide beta (CALCB), have been uncovered in human and rodents, four calcitonin genes (CRSP1, CRSP2, CRSP3 and CALCB) are known in pigs [39]. The associated transcript isoforms can code for numerous neuropeptides. The distinct evolutionary path of the CALCA gene family between pigs and other biomedical species challenges the direct alignment of published associations. Nevertheless, an overarching profile can be identified between the over-expression of the calcitonin transcript isoforms in the hippocampus of MIA relative to control pigs and published work in other species and MIA-related phenotypes. The N-terminal procalcitonin (NPCT) neuropeptide, cleaved from calcitonin producing CALCA transcript isoform, participates in an inflammatory response and is a potential human biomarker and therapeutic target for Alzheimer disease [92]. Also, calcitonin gene-related peptide 1 (alpha-CGRP), a neuropeptide produced from a different CALCA transcript isoform that produces NPCT, plays an important role in social interactions with autism spectrum disorders [92–94].

Significant effects of MIA amygdala transcript isoforms were detected on AVPR1B, DPP4, GNRHR2, growth hormone-releasing hormone receptor (GHRHR), islet amyloid polypeptide (IAPP), OLR1, and POMC. Neuropeptide IAPP is associated with motivated behaviors [85]. Participants in the neuroactive ligand-receptor interaction pathway also detected an association with MIA at the gene level [15]. Similar to PYY, this neuropeptide has been linked to changes in feeding patterns that are also observed in autism spectrum disorder patients with the over-expression of IAPP transcript isoforms in the amygdala of MIA pigs, this protein aggregates in amyloid deposits and the detection of IAPP in the brain has been associated with cognitive decline. Also over-expressed in the amygdala of MIA relative to control pigs was PMCH. This result substantiates reports that dysregulation of PMCH is associated with the development of affective disorders [95].

Among other transcript isoforms dysregulated by MIA in the amygdala, the detection of POMC is in agreement with our analysis at the gene level [15] and with reports of pre- and post-translational bioactive forms associated with behaviors observed in autism spectrum disorders [96]. AVPR1B has been associated with autism spectrum disorders and is a receptor involved in the AVP-mediated activation of the hypothalamic-pituitary-adrenal-axis [97–99]. Consistent with this known function, and AVPR1B transcript isoform was over-expressed in the amygdala of MIA relative to control pigs. The impact of MIA on the expression of a GHRH transcript isoform could relate to the established influence of this hormone neuropeptide in animal cognition [100, 101].

OLR1 (also known as lectin-like oxidized low-density lipoprotein receptor-1, LOX-1) participates in inflammatory responses [102] and agrees with these results OLR1 transcript isoform was over-expressed in the amygdala of MIA relative to control pigs. In the present study, DPP4 is a connecting pathway gene with a transcript isoform under-expressed in the amygdala of MIA relative to control pigs. The observed pattern agrees with the role of DPP4 in processing the neuropeptides substance P and NPY and immune response [103] and the proposed association with autism spectrum disorders [104]. In agreement with the under-expression of the neuropeptide insulin-like growth factor 2 (IGF2) transcript isoform in the amygdala of MIA relative to control pigs, IGF2 treatment can reverse behavior disorders such as anxiety-like phenotypes [105]. IGF2 has also been associated with autism spectrum disorders and similar phenotypes [105, 106].

Bioactive neuropeptides result from multi-tiered pre- and post-transcriptional and translational processes that can influence the profile of neuropeptides across brain regions and should be considered in understanding the impact of MIA on neuropeptide systems. This study focused on exposing the effect of MIA at the post-transcriptional stage. However, pre-transcriptional effects such as epigenetics and post-transcriptional effects such as post-translational modifications can further alter the neuropeptide and receptor system’s profile and activity. Methylation can alter glucocorticoid receptors’ expression in a tissue-specific manner [107]. High-throughput or candidate proteomic detection of neuropeptides is complex. The small size and complex post-translational modifications and differential cleavage in different tissues result in some neuropeptides’ differential occurrence, such as glucagon [108]. Our results demonstrated the impact of MIA on multiple isoform profiles corresponding to genes in the neuropeptide system that can modulate behavior disorders.

## 5. Conclusions

The present study offered additional insights into the impact of MIA on neuropeptide systems by exposing the prolonged effects on transcript isoforms in the hippocampus and amygdala. The analysis of transcript isoforms both uncovered transcription dysregulation that may have been hindered by the analysis of the system at the gene level, and aided in the corroboration of other gene-level findings. The present study detected multiple neuropeptides, neuropeptide receptors. It connected gene transcript isoforms that were significantly differentially expressed in response to the maternal signals elicited in response to infection during gestation.

We confirmed that the present MIA model elicited by viral infection during gestation in pigs assisted in the identification of genes that have known associations with neurodevelopmental disorders, including schizophrenia spectrum disorders, autism spectrum disorders, and Alzheimer’s disease. This study exposed differences in MIA effect on transcript isoforms from the same gene within and across brain regions. This finding suggests that the identification of effective targets to ameliorate MIA and neurodevelopmental disorders necessitate discrimination among transcript isoforms. Our results advance the understanding of proposed neuropeptide-based antipsychotic and antidepressant therapies [109, 110] to ameliorate behavior disorders induced by MIA.

## Supplementary Material

Supplementary Figures

Supplementary Table 1

## Figures and Tables

**Table 1.** Number of neuropeptides, neuropeptide receptors, and connecting and genes differentially expressed (*P*-value < 0.01) between maternal immune activation and control pigs in the hippocampus and amygdala.

Brain Region	Transcript			Gene		

	Neurop	Recep	Connect	Neurop	Recep	Connect

Hippocampus	11	8	8	10	8	7
Amygdala	2	3	2	2	3	2
Overlap	1	1	0	1	1	0

Connect, number of transcript isoforms from connecting pathway transcripts or genes; Neurop, number of transcript isoforms from neuropeptide transcripts or genes; Recep, number of transcript isoforms from neuropeptide receptor transcripts or genes.

**Table 2.** Transcript isoforms over-expressed (*P*-value < 0.01) in MIA relative to control pigs in the hippocampus.

Type	Symbol	Accession	Name	Diff	*P*-Value

Connect	EPB41	XM_021095719.1	erythrocyte membrane protein band 4.1, transcript variant X46	22.29	5.65E-03
Connect	ESR1	XM_021083031.1	estrogen receptor 1, transcript variant X6	11.82	5.66E-06
Connect	PLCG1	XM_021078391.1	phospholipase C gamma 1, transcript variant X4	1.69	5.53E-03
Connect	PLCG1	XM_021078394.1	phospholipase C gamma 1, transcript variant X7	1.63	1.60E-03
Connect	SLA-DQA1	NM_001114062.2	MHC class II histocompatibility antigen SLA-DQA	6.39	7.97E-04
Connect	SRPK2	XM_021102581.1	SRSF protein kinase 2, transcript variant X5	10.28	3.17E-03
Connect	UBQLN1	XM_021064659.1	ubiquilin 1, transcriptvariant X2	1.28	3.40E-03
Neurop	ADCYAP1	XM_021093591.1	adenylate cyclase activating polypeptide 1, transcript variant X2	3.17	2.33E-03
Neurop	CALCB	NM_001102473.1	calcitonin-related polypeptide beta	11.60	7.23E-04
Neurop	CRSP1	XM_021080192.1	calcitonin-related polypeptide beta, transcript variant X3	1.61	5.31E-03
Neurop	IGF1	XM_005664195.3	insulin like growth factor 1, transcript variant X1	1.87	9.83E-03
Neurop	IGF1	XM_005664199.3	insulin like growth factor 1, transcript variant X7	1.53	7.61E-03
Neurop	RLN2	XM_021082276.1	relaxin 2, transcript variant X1	2.86	6.20E-03
Neurop	SCG2	NM_001012299.1	secretogranin II	1.62	6.19E-03
Neurop	TRH	XM_005669803.3	thyrotropin releasing hormone, transcript variant X2	3.05	6.72E-03
Recep	GLP1R	NM_001256594.1	glucagon like peptide 1 receptor	4.75	5.17E-03
Recep	MRGPRX2	XM_013994496.2	MAS related GPR family member X2	2.48	8.52E-03
Recep	NPSR1	XM_021079326.1	neuropeptide S receptor 1	10.66	2.55E-04
Recep	NPY5R	XR_002346654.1	neuropeptide Y receptor Y5, transcript variant X2	1.75	7.10E-03
Recep	OLR1	NM_213805.1	oxidized low density lipoprotein receptor 1	3.93	7.19E-05
Recep	SLC40A1	XM_013984335.2	solute carrier family 40 member 1, transcript variant X2	1.69	1.29E-03

Type: neuropeptide (Neurop), prohormone receptor (Recep) or Connecting pathway gene (Connect). Symbol, Accession, and Name: NCBI gene symbol, transcript accession number and transcript name; Diff: The expected difference between MIA and Control pigs; *P*-Value: the significance of the difference between Control and MIA pigs.

**Table 3.** Transcript isoforms under-expressed (*P*-value < 0.01) in MIA relative to control pigs in the hippocampus.

Type	Symbol	Accession	Name	Diff	*P*-Value

Connect	HSPG2	XM_021095484.1	heparan sulfate proteoglycan 2, transcript variant X5	13.73	8.30E-03
Neurop	AVP	NM_213952.2	arginine vasopressin	2.75	8.07E-03
Neurop	HCRT	NM_214156.2	hypocretin neuropeptide precursor	2.97	3.39E-05
Neurop	PYY	XM_021066091.1	peptide YY, transcript variant X1	2.76	9.85E-05
Neurop	RLN3	XM_013994666.2	relaxin 3, transcript variant X3	1.53	8.07E-04
Recep	ADCYAP1R1	XM_021078471.1	ADCYAP receptor type I, transcript variant X3	32.07	2.72E-07
Recep	GIPR	XM_021094579.1	gastric inhibitory polypeptide receptor, transcript variant X2	3.02	1.89E-03
Recep	NTSR2	XM_021087826.1	neurotensin receptor 2	1.32	1.35E-03

Type: neuropeptide (Neurop), prohormone receptor (Recep) or Connecting pathway gene (Connect). Symbol, Accession, and Name: NCBI gene symbol, transcript accession number and transcript name; Diff: The expected difference between Control and MIA pigs; *P*-Value: the significance of the difference between Control and MIA pigs.

**Table 4.** Transcript isoforms differential expressed in MIA relative to control pigs (*P*-value < 0.01) in the amygdala.

Type	Symbol	Accession	Name	Diff	*P*-Value

Connect	DPP4	NM_214257.1	dipeptidyl peptidase 4	2.19	6.44E-03
Connect	PAM	XM_021084567.1	peptidylglycine alpha-amidating monooxygenase, transcript variant X17	0.14	9.23E-03
Neurop	IAPP	XR_002344089.1	islet amyloid polypeptide, transcript variant X2	0.58	5.91E-03
Neurop	POMC	XM_021085834.1	proopiomelanocortin, transcript variant X1	4.75	1.52E-04
Neurop	PYY	XM_021066091.1	peptide YY, transcript variant X1	3.27	5.82E-04
Recep	AVPR1B	XM_003130445.3	arginine vasopressin receptor 1B	3.47	3.11E-03
Recep	GHRHR	NM_214035.2	growth hormone releasing hormone receptor	4.29	4.07E-03
Recep	GNRHR2	NM_001001639.2	gonadotropin-releasing hormone (type 2) receptor 2	0.54	4.69E-03
Recep	OLR1	NM_213805.1	oxidized low density lipoprotein receptor 1	0.33	3.40E-03

Type: neuropeptide (Neurop), prohormone receptor (Recep) or Connecting pathway gene (Connect). Symbol, Accession, and Name: NCBI gene symbol, transcript accession number and transcript name; Diff: The expected difference between Control and MIA pigs; *P*-Value: the significance of the difference between Control and MIA pigs.

## References

[1] Prins JR, Eskandar S, Eggen BJL, Scherjon SA. Microglia, the missing link in maternal immune activation and fetal neurodevelopment; and a possible link in preeclampsia and disturbed neurodevelopment? Journal of Reproductive Immunology. 2018; 126: 18–22. 29421625 10.1016/j.jri.2018.01.004

[2] Odorizzi PM, Feeney ME. Impact of in utero exposure to malaria on fetal T cell immunity. Trends in Molecular Medicine. 2016; 22: 877–888. 27614925 10.1016/j.molmed.2016.08.005 PMC5048621

[3] Kroismayr R, Baranyi U, Stehlik C, Dorfleutner A, Binder BR, Lipp J. HERC5, a HECT E3 ubiquitin ligase tightly regulated in LPS activated endothelial cells. Journal of Cell Science. 2004; 117: 4749–4756. 15331633 10.1242/jcs.01338

[4] Rutherford KMD, Piastowska-Ciesielska A, Donald RD, Robson SK, Ison SH, Jarvis S, Prenatal stress produces anxiety prone female offspring and impaired maternal behaviour in the domestic pig. Physiology & Behavior. 2014; 129: 255–264. 24631303 10.1016/j.physbeh.2014.02.052

[5] Mattei D, Ivanov A, Ferrai C, Jordan P, Guneykaya D, Buonfiglioli A, Maternal immune activation results in complex microglial transcriptome signature in the adult offspring that is reversed by minocycline treatment. Translational Psychiatry. 2017; 7: e1120–e1120. 28485733 10.1038/tp.2017.80 PMC5534948

[6] Canetta S, Bolkan S, Padilla-Coreano N, Song LJ, Sahn R, Harrison NL, Maternal immune activation leads to selective functional deficits in offspring parvalbumin interneurons. Molecular Psychiatry. 2016; 21: 956–968. 26830140 10.1038/mp.2015.222 PMC4914410

[7] Knuesel I, Chicha L, Britschgi M, Schobel SA, Bodmer M, Hellings JA, Maternal immune activation and abnormal brain development across CNS disorders. Nature Reviews: Neurology. 2014; 10: 643–660. 25311587 10.1038/nrneurol.2014.187

[8] Burbach JPH. What are neuropeptides? Methods in Molecular Biology. 2011; 789: 1–36. 21922398 10.1007/978-1-61779-310-3_1

[9] Hökfelt T, Bartfai T, Bloom F. Neuropeptides: opportunities for drug discovery. Te Lancet Neurology. 2003; 2: 463–472. 10.1016/s1474-4422(03)00482-4 12878434

[10] Mendel HC, Kaas Q, Muttenthaler M. Neuropeptide signalling systems-an underexplored target for venom drug discovery. Biochemical Pharmacology. 2020; 181: 114129. 32619425 10.1016/j.bcp.2020.114129 PMC7116218

[11] Smith SJ, Hawrylycz M, Rossier J, Sümbül U. New light on cortical neuropeptides and synaptic network plasticity. Current Opinion in Neurobiology. 2020; 63: 176–188. 32679509 10.1016/j.conb.2020.04.002

[12] Fricker LD. Neuropeptide-processing enzymes: applications for drug discovery. The AAPS Journal. 2005; 7: E449–E455. 16353923 10.1208/aapsj070244 PMC2750981

[13] Marotta R, Risoleo MC, Messina G, Parisi L, Carotenuto M, Vetri L, The neurochemistry of autism. Brain Sciences. 2020; 10: 163. 10.3390/brainsci10030163 PMC7139720 32182969

[14] Autio J, Stenback V, Gagnon DD, Leppaluoto J, Herzig KH. (Neuro) Peptides, physical activity, and cognition. Journal of Clinical Medicine. 2020; 9: 2592. 10.3390/jcm9082592 PMC7464334 32785144

[15] Keever MR, Zhang P, Bolt CR, Antonson AM, Rymut HE, Caputo MP, Lasting and sex-dependent impact of maternal immune activation on molecular pathways of the amygdala. Frontiers in Neuroscience. 2020; 14: 774. 32848554 10.3389/fnins.2020.00774 PMC7431923

[16] Southey BR, Romanova EV, Rodriguez-Zas SL, Sweedler JV. Bioinformatics for prohormone and neuropeptide discovery. Methods in Molecular Biology. 2018; 38: 71–96. 10.1007/978-1-4939-7537-2_5 PMC5847276 29476505

[17] Southey BR, Sweedler JV, Rodriguez-Zas SL. A python analytical pipeline to identify prohormone precursors and predict prohormone cleavage sites. Frontiers in Neuroinformatics. 2008; 2: 7. 19169350 10.3389/neuro.11.007.2008 PMC2610252

[18] Gothard KM. Multidimensional processing in the amygdala. Nature Reviews Neuroscience. 2020; 21: 565–575. 32839565 10.1038/s41583-020-0350-y PMC7714370

[19] Nikolenko VN, Oganesyan MV, Rizaeva NA, Kudryashova VA, Nikitina AT, Pavliv MP, Amygdala: neuroanatomical and morphophysiological features in terms of neurological and neurodegenerative diseases. Brain Sciences. 2020; 10: 502. 10.3390/brainsci10080502 PMC7465610 32751957

[20] DeMayo MM, Young LJ, Hickie IB, Song YJC, Guastella AJ. Circuits for social learning: a unified model and application to Autism Spectrum Disorder. Neuroscience & Biobehavioral Reviews. 2019; 107: 388–398. 31560922 10.1016/j.neubiorev.2019.09.034 PMC6875617

[21] Goodrich M, Armour AC, Panchapakesan K, You X, Devaney J, Knoblach S, PAC1R genotype to phenotype correlations in autism spectrum disorder. Autism Research. 2019; 12: 200–211. 30556326 10.1002/aur.2051 PMC6665682

[22] Meurens F, Summerfield A, Nauwynck H, Saif L, Gerdts V. The pig: a model for human infectious diseases. Trends in Microbiology. 2012; 20: 50–57. 22153753 10.1016/j.tim.2011.11.002 PMC7173122

[23] Enjuanes L, Gorbalenya AE, de Groot RJ, Cowley JA, Ziebuhr J, Snijder EJ. Nidovirales. In Encyclopedia of Virology (pp. 419–430). 3rd Edition. Mahy BWJ, Van Regenmortel MHV, editors. Oxford: Academic Press. 2008.

[24] Lunney JK, Benfield DA, Rowland RRR. Porcine reproductive and respiratory syndrome virus: an update on an emerging and reemerging viral disease of swine. Virus Research. 2010; 154: 1–6. 20951175 10.1016/j.virusres.2010.10.009 PMC7172856

[25] Rymut HE, Bolt CR, Caputo MP, Houser AK, Antonson AM, Zimmerman JD, Long-lasting impact of maternal immune activation and interaction with a second immune challenge on pig behavior. Frontiers in Veterinary Science. 2020; 7: 561151. 33330688 10.3389/fvets.2020.561151 PMC7732429

[26] Félix B, Léger M, Albe-Fessard D, Marcilloux JC, Rampin O, Laplace JP. Stereotaxic atlas of the pig brain. Brain Research Bulletin. 1999; 49: 1–137. 10466025 10.1016/s0361-9230(99)00012-x

[27] Zhang P, Southey BR, Sweedler JV, Pradhan A, S.L. R-Z. Enhanced understanding of molecular interactions and function underlying pain processes through networks of transcript isoforms, genes, and gene families. Advances and Applications in Bioinformatics and Chemistry. 2021; 14: 49–69. 33633454 10.2147/AABC.S284986 PMC7901473

[28] Bray NL, Pimentel H, Melsted P, Pachter L. Near-optimal probabilistic RNA-seq quantification. Nature Biotechnology. 2016; 34: 525–527. 10.1038/nbt.3519 27043002

[29] Martin A, Ochagavia ME, Rabasa LC, Miranda J, Fernandez-de-Cossio J, Bringas R. BisoGenet: a new tool for gene network building, visualization and analysis. BMC Bioinformatics. 2010; 11: 91. 20163717 10.1186/1471-2105-11-91 PMC3098113

[30] Shannon P, Markiel A, Ozier O, Baliga NS, Wang JT, Ramage D, Cytoscape: a software environment for integrated models of biomolecular interaction networks. Genome Research. 2003; 13: 2498–2504. 14597658 10.1101/gr.1239303 PMC403769

[31] Xuan IC, Hampson DR. Gender-dependent effects of maternal immune activation on the behavior of mouse offspring. PLoS ONE. 2014; 9: e104433. 25111339 10.1371/journal.pone.0104433 PMC4128679

[32] Malkova NV, Yu CZ, Hsiao EY, Moore MJ, Patterson PH. Maternal immune activation yields offspring displaying mouse versions of the three core symptoms of autism. Brain, Behavior, and Immunity. 2012; 26: 607–616. 10.1016/j.bbi.2012.01.011 PMC3322300 22310922

[33] Aavani T, Rana SA, Hawkes R, Pittman QJ. Maternal immune activation produces cerebellar hyperplasia and alterations in motor and social behaviors in male and female mice. The Cerebellum. 2015; 14: 491–505. 25863812 10.1007/s12311-015-0669-5

[34] Fair T The contribution of the maternal immune system to the establishment of pregnancy in cattle. Frontiers in Immunology. 2015; 6: 7. 25674085 10.3389/fimmu.2015.00007 PMC4309202

[35] Boulanger-Bertolus J, Pancaro C, Mashour GA. Increasing role of maternal immune activation in neurodevelopmental disorders. Frontiers in Behavioral Neuroscience. 2018; 12: 230. 30344483 10.3389/fnbeh.2018.00230 PMC6182081

[36] Antonson AM, Radlowski EC, Lawson MA, Rytych JL, Johnson RW. Maternal viral infection during pregnancy elicits anti-social behavior in neonatal piglet offspring independent of postnatal microglial cell activation. Brain, Behavior, and Immunity. 2017; 59: 300–312. 10.1016/j.bbi.2016.09.019 27650113

[37] Antonson A, Balakrishnan B, Radlowski E, Petr G, Johnson R. Altered hippocampal gene expression and morphology in fetal piglets following maternal respiratory viral infection. Developmental Neuroscience. 2018; 40: 104–119. 29539630 10.1159/000486850

[38] Tegge AN, Southey BR, Sweedler JV, Rodriguez-Zas SL. Comparative analysis of neuropeptide cleavage sites in human, mouse, rat, and cattle. Mammalian Genome. 2008; 19: 106–120. 18213482 10.1007/s00335-007-9090-9

[39] Porter KI, Southey BR, Sweedler JV, Rodriguez-Zas SL. First survey and functional annotation of prohormone and convertase genes in the pig. BMC Genomics. 2012; 13: 582. 23153308 10.1186/1471-2164-13-582 PMC3499383

[40] Fagerberg L, Hallström BM, Oksvold P, Kampf C, Djureinovic D, Odeberg J, Analysis of the human tissue-specific expression by genome-wide integration of transcriptomics and antibody-based proteomics. Molecular & Cellular Proteomics. 2014; 13: 397–406. 10.1074/mcp.M113.035600 PMC3916642 24309898

[41] Fetissov SO, Averina OV, Danilenko VN. Neuropeptides in the microbiota-brain axis and feeding behavior in autism spectrum disorder. Nutrition. 2019; 61: 43–48. 30684851 10.1016/j.nut.2018.10.030

[42] Larhammar D Evolution of neuropeptide Y, peptide YY and pancreatic polypeptide. Regulatory Peptides. 1996; 62: 1–11. 8738876 10.1016/0167-0115(95)00169-7

[43] McBride WJ, Kimpel MW, McClintick JN, Ding Z, Hyytia P, Colombo G, Gene expression within the extended amygdala of 5 pairs of rat lines selectively bred for high or low ethanol consumption. Alcohol. 2013; 47: 517–529. 24157127 10.1016/j.alcohol.2013.08.004 PMC3866700

[44] Kwon H, Mackie DI, Bonnavion R, Mercier AL, Helker CSM, Son T, The orphan G-protein coupled receptor 182 is a negative regulator of definitive hematopoiesis through leukotriene B4 signaling. ACS Pharmacology & Translational Science. 2020; 3: 676–689. 32832870 10.1021/acsptsci.0c00020 PMC7432686

[45] Jo-Watanabe A, Okuno T, Yokomizo T. The role of leukotrienes as potential therapeutic targets in allergic disorders. International Journal of Molecular Sciences. 2019; 20: 3580. 10.3390/ijms20143580 PMC6679143 31336653

[46] Harville T, Rhodes-Clark B, Bennuri SC, Delhey L, Slattery J, Tippett M, Inheritance of HLA-Cw7 associated with autism spectrum disorder (ASD). Frontiers in Psychiatry. 2019; 10: 612. 31572230 10.3389/fpsyt.2019.00612 PMC6749146

[47] Rossignol DA, Frye RE. A review of research trends in physiological abnormalities in autism spectrum disorders: immune dysregulation, inflammation, oxidative stress, mitochondrial dysfunction and environmental toxicant exposures. Molecular Psychiatry. 2012; 17: 389–401. 22143005 10.1038/mp.2011.165 PMC3317062

[48] Katafuchi T, Hamano K, Kikumoto K, Minamino N. Isolation and characterization of a glycine-extended form of calcitonin receptor-stimulating peptide-1: another biologically active form of calcitonin receptor-stimulating peptide-1. Peptides. 2005; 26: 2616–2623. 16023259 10.1016/j.peptides.2005.06.004

[49] Aguilar-Valles A, Flores C, Luheshi GN. Prenatal inflammation-induced hypoferremia alters dopamine function in the adult offspring in rat: relevance for schizophrenia. PLoS ONE. 2010; 5: e10967. 20532043 10.1371/journal.pone.0010967 PMC2881043

[50] Carter CJ. Schizophrenia: a pathogenetic autoimmune disease caused by viruses and pathogens and dependent on genes. Journal of Pathogens. 2011; 2011: 128318. 22567321 10.4061/2011/128318 PMC3335463

[51] Kim HY, Yang YR, Hwang H, Lee HE, Jang HJ, Kim J, Deletion of PLCgamma1 in GABAergic neurons increases seizure susceptibility in aged mice. Scientific Reports. 2019; 9: 17761. 31780806 10.1038/s41598-019-54477-4 PMC6882884

[52] Hashimoto T, Arion D, Unger T, Maldonado-Avilés JG, Morris HM, Volk DW, Alterations in GABA-related transcriptome in the dorsolateral prefrontal cortex of subjects with schizophrenia. Molecular Psychiatry. 2008; 13: 147–161. 17471287 10.1038/sj.mp.4002011 PMC2882638

[53] Mabes ES, Moraczewski J, Chishom T, Dymanus K, Linder D, Yu JC. Discovery of an association between influenza infection rates and the incidences of craniosynostosis in the United States: a potentially modifiable risk factor. FACE. 2020; 1: 97–104.

[54] van der Meer D, Hoekstra PJ, van Donkelaar M, Bralten J, Oosterlaan J, Heslenfeld D, Predicting attention-deficit/hyperactivity disorder severity from psychosocial stress and stress-response genes: a random forest regression approach. Translational Psychiatry. 2017; 7: e1145–e1145. 28585928 10.1038/tp.2017.114 PMC5537639

[55] Laas K, Eensoo D, Paaver M, Lesch K, Reif A, Harro J. Further evidence for the association of the NPSR1 gene a/T polymorphism (Asn107Ile) with impulsivity and hyperactivity. Journal of Psychopharmacology. 2015; 29: 878–883. 25744621 10.1177/0269881115573803

[56] Hong Y, Chan CB, Kwon I-, Li X, Song M, Lee H-, SRPK2 phosphorylates tau and mediates the cognitive defects in Alzheimer’s disease. Journal of Neuroscience. 2012; 32: 17262–17272. 23197718 10.1523/JNEUROSCI.3300-12.2012 PMC3518045

[57] Bonora E, Lamb JA, Barnby G, Sykes N, Moberly T, Beyer KS, Mutation screening and association analysis of six candidate genes for autism on chromosome 7q. European Journal of Human Genetics. 2005; 13: 198–207. 15523497 10.1038/sj.ejhg.5201315

[58] Doi H, Fujisawa TX, Iwanaga R, Matsuzaki J, Kawasaki C, Tochigi M, Association between single nucleotide polymorphisms in estrogen receptor 1/2 genes and symptomatic severity of autism spectrum disorder. Research in Developmental Disabilities. 2018; 82: 20–26. 29526366 10.1016/j.ridd.2018.02.014

[59] Pinsonneault JK, Frater JT, Kompa B, Mascarenhas R, Wang D, Sadee W. Intronic SNP in ESR1 encoding human estrogen receptor alpha is associated with brain ESR1 mRNA isoform expression and behavioral traits. PLoS ONE. 2017; 12: e0179020. 28617822 10.1371/journal.pone.0179020 PMC5472281

[60] Lorenzen E, Dodig-Crnkovic T, Kotliar IB, Pin E, Ceraudo E, Vaughan RD, Multiplexed analysis of the secretin-like GPCR-RAMP interactome. Science Advances. 2019; 5: eaaw2778. 31555726 10.1126/sciadv.aaw2778 PMC6750928

[61] Couvineau A, Laburthe M. VPAC receptors: structure, molecular pharmacology and interaction with accessory proteins. British Journal of Pharmacology. 2012; 166: 42–50. 21951273 10.1111/j.1476-5381.2011.01676.x PMC3415636

[62] Shen S, Gehlert DR, Collier DA. PACAP and PAC1 receptor in brain development and behavior. Neuropeptides. 2013; 47: 421–430. 24220567 10.1016/j.npep.2013.10.005

[63] Liao C, de Molliens MP, Schneebeli ST, Brewer M, Song G, Chatenet D, Targeting the PAC1 receptor for neurological and metabolic disorders. current topics in medicinal chemistry. 2019; 19: 1399–1417. 31284862 10.2174/1568026619666190709092647 PMC6761004

[64] Abad C, Tan Y. Immunomodulatory roles of PACAP and VIP: lessons from knockout mice. Journal of Molecular Neuroscience. 2018; 66: 102–113. 30105629 10.1007/s12031-018-1150-y

[65] Shintani N, Hashimoto H, Tanaka K, Kawagishi N, Kawaguchi C, Hatanaka M, Serotonergic inhibition of intense jumping behavior in mice lacking PACAP (Adcyap1−/−). Annals of the New York Academy of Sciences. 2006; 1070: 545–549. 16888223 10.1196/annals.1317.079

[66] Otto C, Martin M, Paul Wolfer D, Lipp H, Maldonado R, Schütz G. Altered emotional behavior in PACAP-type-I-receptor-deficient mice. Molecular Brain Research. 2001; 92: 78–84. 11483244 10.1016/s0169-328x(01)00153-x

[67] Nicot A Altered social behavior in pituitary adenylate cyclase-activating polypeptide type I receptor-deficient mice. Journal of Neuroscience. 2004; 24: 8786–8795. 15470144 10.1523/JNEUROSCI.1910-04.2004 PMC6729943

[68] Blechman J, Levkowitz G. Alternative splicing of the pituitary adenylate cyclase-activating polypeptide receptor PAC1: mechanisms of fine tuning of brain activity. Frontiers in Endocrinology. 2013; 4: 55. 23734144 10.3389/fendo.2013.00055 PMC3659299

[69] Hammack SE, May V. Pituitary adenylate cyclase activating polypeptide in stress-related disorders: data convergence from animal and human studies. Biological Psychiatry. 2015; 78: 167–177. 25636177 10.1016/j.biopsych.2014.12.003 PMC4461555

[70] Ushiyama M, Ikeda R, Yoshida M, Mori K, Kangawa K, Sugawara H, Alternative splicing of the pituitary adenylate cyclase-activating polypetide (PACAP) receptor contributes to function of PACAP-27. Journal of Molecular Neuroscience. 2010; 42: 341–348. 20473586 10.1007/s12031-010-9385-2

[71] Amir-Zilberstein L, Blechman J, Sztainberg Y, Norton WJ, Reuveny A, Borodovsky N, Homeodomain protein otp and activity-dependent splicing modulate neuronal adaptation to stress. Neuron. 2012; 73: 279–291. 22284183 10.1016/j.neuron.2011.11.019 PMC4387198

[72] Biran J, Gliksberg M, Shirat I, Swaminathan A, Levitas-Djerbi T, Appelbaum L, Splice-specific deficiency of the PTSD-associated gene PAC1 leads to a paradoxical age-dependent stress behavior. Scientific Reports. 2020; 10: 9559. 32533011 10.1038/s41598-020-66447-2 PMC7292827

[73] King SB, Toufexis DJ, Hammack SE. Pituitary adenylate cyclase activating polypeptide (PACAP), stress, and sex hormones. Stress. 2017; 20: 465–475. 28610473 10.1080/10253890.2017.1336535 PMC6724739

[74] Miles OW, May V, Hammack SE. Pituitary adenylate cyclase-activating peptide (PACAP) signaling and the dark side of addiction. Journal of Molecular Neuroscience. 2019; 68: 453–464. 30074172 10.1007/s12031-018-1147-6 PMC6732790

[75] Krishna DBA, Yang N, Romanova EV, Rubakhin SS, Tipton A, Dripps I, PACAP and other neuropeptide targets link chronic migraine and opioid-induced hyperalgesia in mouse models. Molecular & Cellular Proteomics. 2019; 18: 2447–2458. 31649062 10.1074/mcp.RA119.001767 PMC6885698

[76] Tan Y, Waschek JA. Targeting VIP and PACAP receptor signalling: new therapeutic strategies in multiple sclerosis. American Society for Neurochemistry. 2011; 3: AN20110024. 10.1042/AN20110024 PMC3189630 21895607

[77] Denes V, Geck P, Mester A, Gabriel R. Pituitary adenylate cyclase-activating polypeptide: 30 years in research spotlight and 600 million years in service. Journal of Clinical Medicine. 2019; 8: 1488. 10.3390/jcm8091488 PMC6780647 31540472

[78] Fang Y, Ren R, Shi H, Huang L, Lenahan C, Lu Q, Pituitary adenylate cyclase-activating polypeptide: a promising neuroprotective peptide in stroke. Aging and Disease. 2020; 11: 1496. 33269103 10.14336/AD.2020.0626 PMC7673855

[79] Otto C, Kovalchuk Y, Wolfer DP, Gass P, Martin M, Zuschratter W, Impairment of mossy fiber long-term potentiation and associative learning in pituitary adenylate cyclase activating polypeptide type I receptor-deficient mice. The Journal of Neuroscience. 2001; 21: 5520–5527. 11466423 10.1523/JNEUROSCI.21-15-05520.2001 PMC6762677

[80] Bousquet-Moore D, Mains RE, Eipper BA. Peptidylgycine alpha-amidating monooxygenase and copper: a gene-nutrient interaction critical to nervous system function. Journal of Neuroscience Research. 2010; 88: 2535–2545. 20648645 10.1002/jnr.22404 PMC3732055

[81] Yin P, Bousquet-Moore D, Annangudi SP, Southey BR, Mains RE, Eipper BA, Probing the production of amidated peptides following genetic and dietary copper manipulations. PLoS ONE. 2011; 6: e28679. 22194882 10.1371/journal.pone.0028679 PMC3241674

[82] Fatemi SH, Folsom TD, Reutiman TJ, Sidwell RW. Viral regulation of aquaporin 4, connexin 43, microcephalin and nucleolin. Schizophrenia Research. 2008; 98: 163–177. 17997079 10.1016/j.schres.2007.09.031 PMC2259220

[83] Zettergren A, Jonsson L, Johansson D, Melke J, Lundström S, Anckarsäter H, Associations between polymorphisms in sex steroid related genes and autistic-like traits. Psychoneuroendocrinology. 2013; 38: 2575–2584. 23867117 10.1016/j.psyneuen.2013.06.004

[84] Kumar JR, Rajkumar R, Jayakody T, Marwari S, Hong JM, Ma S, Relaxin’ the brain: a case for targeting the nucleus incertus network and relaxin-3/RXFP3 system in neuropsychiatric disorders. British Journal of Pharmacology. 2017; 174: 1061–1076. 27597467 10.1111/bph.13564 PMC5406295

[85] Kern KA, Mietlicki-Baase EG. Distributed amylin receptor signaling and its influence on motivated behavior. Physiology & Behavior. 2020; 222: 112958. 32439326 10.1016/j.physbeh.2020.112958

[86] Stanciu GD, Bild V, Ababei DC, Rusu RN, Cobzaru A, Paduraru L, Link between diabetes and Alzheimer’s disease due to the shared amyloid aggregation and deposition involving both neurodegenerative changes and neurovascular damages. Journal of Clinical Medicine. 2020; 9: 1713. 10.3390/jcm9061713 PMC7357086 32503113

[87] Steinman G IGF-autism prevention/amelioration. Medical Hypotheses. 2019; 122: 45–47. 30593420 10.1016/j.mehy.2018.10.015

[88] Linker SB, Mendes APD, Marchetto MC. IGF-1 treatment causes unique transcriptional response in neurons from individuals with idiopathic autism. Molecular Autism. 2020; 11: 55. 32591005 10.1186/s13229-020-00359-w PMC7320548

[89] Riikonen R Treatment of autistic spectrum disorder with insulin-like growth factors. European Journal of Paediatric Neurology. 2016; 20: 816–823. 27562096 10.1016/j.ejpn.2016.08.005

[90] Jameel MN, Li Q, Mansoor A, Qiang X, Sarver A, Wang X, Long-term functional improvement and gene expression changes after bone marrow-derived multipotent progenitor cell transplantation in myocardial infarction. American Journal of Physiology-Heart and Circulatory Physiology. 2010; 298: H1348–H1356. 20173039 10.1152/ajpheart.01100.2009 PMC3774483

[91] Theoharides TC, Tsilioni I. Autism spectrum disorders. In Neuroimmune Pharmacology (pp. 643–659). Springer. 2017.

[92] Tavares E, Antequera D, López-González I, Ferrer I, Miñano FJ, Carro E. Potential role of aminoprocalcitonin in the pathogenesis of alzheimer disease. The American Journal of Pathology. 2016; 186: 2723–2735. 27497681 10.1016/j.ajpath.2016.06.006

[93] McNamara IM, Borella AW, Bialowas LA, Whitaker-Azmitia PM. Further studies in the developmental hyperserotonemia model (DHS) of autism: social, behavioral and peptide changes. Brain Research. 2008; 1189: 203–214. 18062943 10.1016/j.brainres.2007.10.063

[94] Yang CJ, Tan HP, Du YJ. The developmental disruptions of serotonin signaling may involved in autism during early brain development. Neuroscience. 2014; 267: 1–10. 24583042 10.1016/j.neuroscience.2014.02.021

[95] García-Fuster MJ, Parks GS, Clinton SM, Watson SJ, Akil H, Civelli O. The melanin-concentrating hormone (MCH) system in an animal model of depression-like behavior. European Neuropsychopharmacology. 2012; 22: 607–613. 22209364 10.1016/j.euroneuro.2011.12.001 PMC3319808

[96] Harno E, Gali Ramamoorthy T, Coll AP, White A. POMC: the physiological power of hormone processing. Physiological Reviews. 2018; 98: 2381–2430. 30156493 10.1152/physrev.00024.2017 PMC6170974

[97] Dempster EL, Burcescu I, Wigg K, Kiss E, Baji I, Gadoros J, Further genetic evidence implicates the vasopressin system in childhood-onset mood disorders. European Journal of Neuroscience. 2009; 30: 1615–1619. 10.1111/j.1460-9568.2009.06930.x 19821843

[98] Dempster EL. Evidence of an association between the vasopressin V1b receptor gene (AVPR1B) and childhood-onset mood disorders. Archives of General Psychiatry. 2007; 64: 1189. 17909131 10.1001/archpsyc.64.10.1189

[99] Cataldo I, Azhari A, Esposito G. A review of oxytocin and arginine-vasopressin receptors and their modulation of autism spectrum disorder. Frontiers in Molecular Neuroscience. 2018; 11: 27. 29487501 10.3389/fnmol.2018.00027 PMC5816822

[100] Jaszberenyi M, Rick FG, Szalontay L, Block NL, Zarandi M, Cai R, Beneficial effects of novel antagonists of GHRH in different models of Alzheimer’s disease. Aging. 2012; 4: 755–767. 23211425 10.18632/aging.100504 PMC3560443

[101] Granata R Peripheral activities of growth hormone-releasing hormone. Journal of Endocrinological Investigation. 2016; 39: 721–727. 26891937 10.1007/s40618-016-0440-x

[102] Ding Z, Liu S, Wang X, Khaidakov M, Fan Y, Deng X, Lectin-like oxidized low-density lipoprotein receptor-1 regulates autophagy and Toll-like receptor 4 in the brain of hypertensive mice. Journal of Hypertension. 2015; 33: 525–533. 25380158 10.1097/HJH.0000000000000411

[103] Waumans Y, Baerts L, Kehoe K, Lambeir AM, De Meester I. The dipeptidyl peptidase family, prolyl oligopeptidase, and prolyl carboxypeptidase in the immune system and inflammatory disease, including atherosclerosis. Frontiers in Immunology. 2015; 6: 387. 26300881 10.3389/fimmu.2015.00387 PMC4528296

[104] Jarmolowska B, Bukalo M, Fiedorowicz E, Cieslinska A, Kordulewska NK, Moszynska M, Role of milk-derived opioid peptides and proline dipeptidyl peptidase-4 in autism spectrum disorders. Nutrients. 2019; 11: 87. 10.3390/nu11010087 PMC6356206 30621149

[105] Steinmetz AB, Stern SA, Kohtz AS, Descalzi G, Alberini CM. Insulin-like growth factor II targets the mTOR pathway to reverse autism-like phenotypes in mice. The Journal of Neuroscience. 2018; 38: 1015–1029. 29217683 10.1523/JNEUROSCI.2010-17.2017 PMC5783959

[106] Mills JL, Hediger ML, Molloy CA, Chrousos GP, Manning-Courtney P, Yu KF, Elevated levels of growth-related hormones in autism and autism spectrum disorder. Clinical Endocrinology. 2007; 67: 230–237. 17547689 10.1111/j.1365-2265.2007.02868.x

[107] Cushing B, Kramer K. Mechanisms underlying epigenetic effects of early social experience: the role of neuropeptides and steroids. Neuroscience & Biobehavioral Reviews. 2005; 29: 1089–1105. 16099507 10.1016/j.neubiorev.2005.04.001

[108] Dey A, Lipkind GM, Rouillé Y, Norrbom C, Stein J, Zhang C, Significance of prohormone convertase 2, PC2, mediated initial cleavage at the proglucagon interdomain site, Lys70-Arg71, to generate glucagon. Endocrinology. 2005; 146: 713–727. 15528303 10.1210/en.2004-1118

[109] Rotzinger S, Lovejoy DA, Tan LA. Behavioral effects of neuropeptides in rodent models of depression and anxiety. Peptides. 2010; 31: 736–756. 20026211 10.1016/j.peptides.2009.12.015

[110] Doosti M-H, Bakhtiari A, Zare P, Amani M, Majidi-Zolbanin N, Babri S, Impacts of early intervention with fluoxetine following early neonatal immune activation on depression-like behaviors and body weight in mice. Progress in NeuroPsychopharmacology and Biological Psychiatry. 2013; 43: 55–65. 10.1016/j.pnpbp.2012.12.003 23270703

