# Supplementary figures and images for: Effects of maternal immune activation in porcine transcript isoforms of neuropeptide and receptor genes
Source: J Integr Neurosci. Author manuscript; Available in PMC 2021 May 7. (PMC8103820; doi:10.31083/j.jin.2021.01.332)

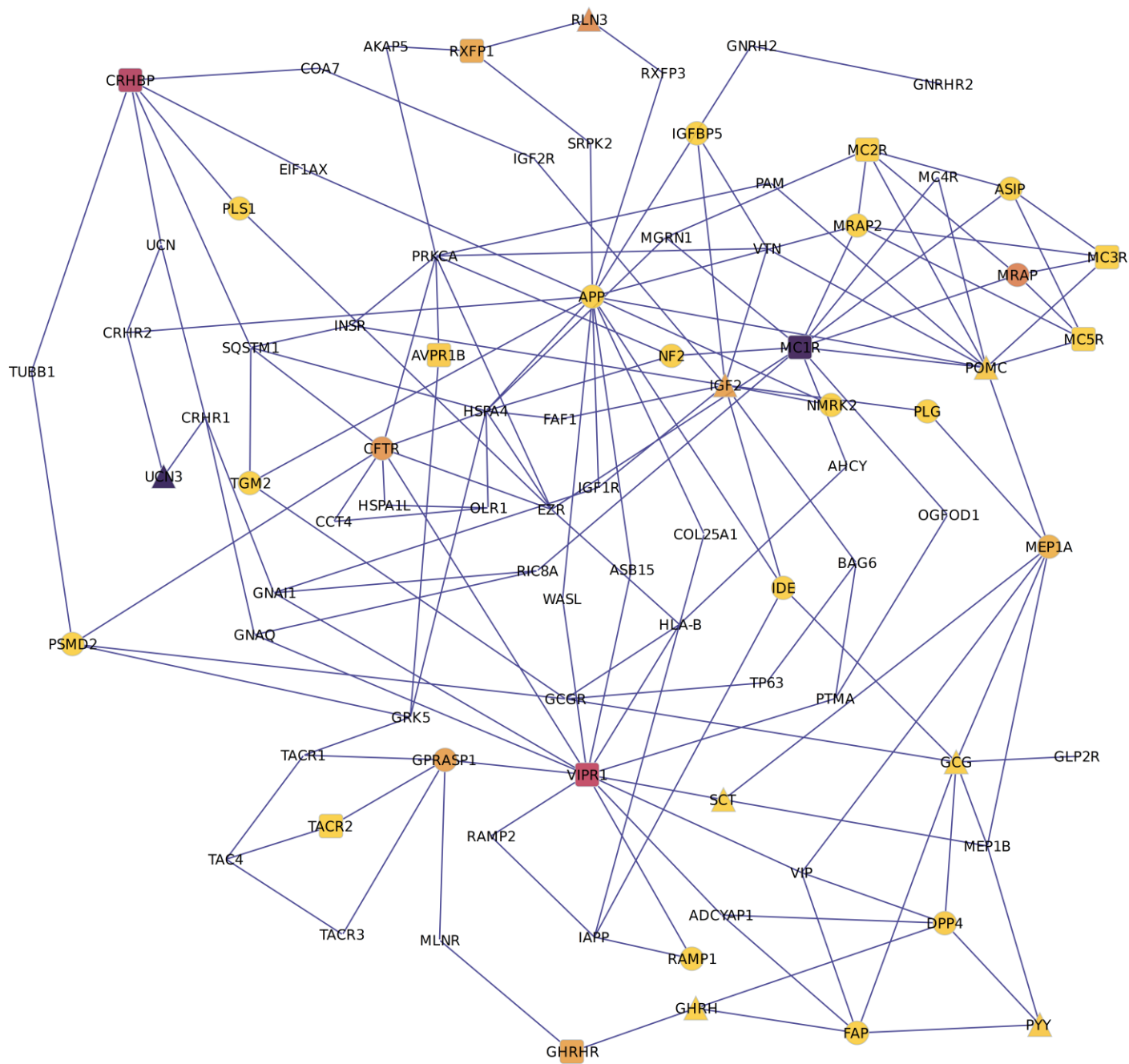

Fig. S1

Fig. S2

Supplement: Supplementary Figures [file NIHMS1693539-supplement-Supplementary_Figures.pdf]
